# An ex vivo comparison of jejunal transection angles and the effect on lumen diameter following end‐to‐end jejunojejunal anastomoses

**DOI:** 10.1111/vsu.14294

**Published:** 2025-06-17

**Authors:** Christopher M. Baldwin, Alexandra Gillen

**Affiliations:** ^1^ Department of Equine Clinical Science Institute of Veterinary Science, University of Liverpool, Leahurst Campus Neston UK

## Abstract

**Objective:**

To evaluate three different jejunal transection angles for end‐to‐end jejunojejunostomies, comparing construction time, lumen size, and suture number.

**Study design:**

Ex vivo cadaver study.

**Sample population:**

Eight euthanized horses each had three mid‐jejunal specimens harvested.

**Methods:**

Jejunal segments were assigned to one of three groups (A30, A45, and A60) and transected at 30, 45, or 60°, respectively. Following transection, a standardized handsewn single‐layer interrupted modified Lembert anastomosis was performed, and construction time and number of sutures placed were recorded. Anastomotic index (AI) was calculated. Statistical analysis was performed using a one‐way ANOVA. Statistical significance was set at *p* < .05.

**Results:**

Transected jejunal lengths increased as the transection angle decreased (*p* < .001). Anastomoses construction time was not different between groups (*p* = .301). The number of sutures required to complete the anastomosis was higher (*p* = .026) for A30 compared to A60 but was not different between A60 and A45 or A45 and A30 (*p* > .333). Mean AI were 93.37, 114.29, and 135.07 for groups A60, A45, and A30 and the AI increased as the transection angle decreased (*p* < .001).

**Conclusion:**

A 60° transection angle reduced lumen size at the anastomosis but a 45 and 30° transection angle resulted in an increased lumen size at the anastomosis. The 45° angle did not increase the number of sutures required. The 30° angle significantly increased the number of sutures required.

**Clinical significance:**

A 45° angle of transection does not result in increased surgical time or number of sutures placed. Further investigation is required to determine the ideal angle of transection.

## 1 INTRODUCTION

Small intestinal disease accounts for 25%–64% of equine colic cases.^1^ Strangulating lesions account for 58%–85% of small intestinal conditions, often requiring a form of resection and anastomosis.^1^ Intestinal anastomoses should ideally create a water‐tight seal, minimize luminal reduction, minimize mucosal eversion, be time efficient and minimize the number of sutures required.^1^, ^2^ During a traditional end‐to‐end resection and anastomosis, the small intestine is transected at 60° from the mesenteric attachment to create a large stoma and preserve blood flow to the antimesenteric border.^1^, ^3^, ^4^, ^5^, ^6^, ^7^, ^8^, ^9^ The 60° recommendation has not been evaluated and the authors clinically perform jejunal transection with a decreased (less than 60°) transection angle to increase anastomosis lumen size.

Surgical methods for tissue apposition have been evaluated, assessing the effect on construction time, lumen size, postoperative complications and survival^3^, ^4^, ^5^, ^6^, ^7^, ^8^, ^9^, ^10^, ^11^, ^12^, ^13^, ^14^ demonstrating that anastomosis construction impacts lumen size, an important factor in postoperative outcome. A large lumen size at the anastomosis reduces the likelihood of impaction at the anastomosis thus potentially reducing the incidence of postoperative colic or postoperative reflux.^1^ Ex vivo studies have demonstrated that all anastomosis patterns reduce lumen size by 15%–45% compared with adjacent control segments,^3^, ^6^, ^7^, ^15^, ^16^, ^17^ with the exception of one report that showed an increase in lumen size ex vivo but a reduction in lumen size in vivo.^4^ Of some concern is the possibility that a more severe reduction in lumen size may develop postoperatively^4^ as a result of muscle tone,^18^ tissue swelling and inflammation,^19^, ^20^ and later fibrosis.^2^, ^10^, ^20^

While the method of tissue apposition at the anastomosis site has been well studied, the angle of tissue transection and its effect on lumen size has not been evaluated. This represents a critical step in the construction of the anastomosis. One potential method of increasing lumen size at the anastomosis site is to perform a wider degree of transection. Our objectives were to evaluate the effect of three different jejunal transection angles for end‐to‐end jejunojejunostomies comparing, construction time, lumen size, and suture number. We hypothesized that anastomosis construction time, number of sutures placed, and anastomosis lumen size would all increase as the transection angle decreased.

## 2 MATERIALS AND METHODS

Currently, there are no studies evaluating the effect of jejunal transection angle on the anastomotic index. This therefore precluded performing a power calculation using effect size in the standard way. Stata^a^ (Intercooled Stata 19.0, StataCorp LLC, College Station, Texas) was therefore utilized to calculate sample size. Using an alpha of 0.05 and an effect size of 0.8 (Cohen's D, large effect size), seven samples per group (i.e., 7 horses) were required to achieve a statistical power of 80%. An additional cadaver was available and therefore eight horses were enrolled in the study.

**FIGURE 1:**
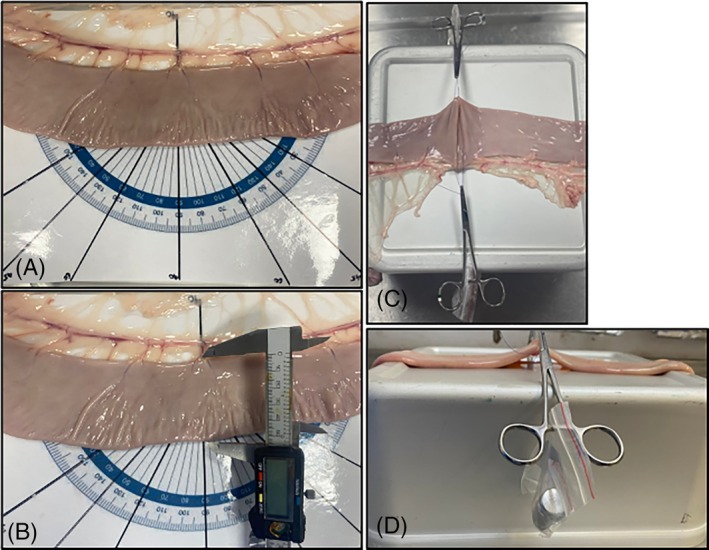
Set‐up of the cadaver material. (A) The jejunal section is laid over the elevated steel tray over a protractor with the 60, 45 and 30° lines highlighted to guide the placement of the Doyen bowel clamps. (B) The mesenteric to anti‐mesenteric length was measured with Vernier calipers. (C and D) Following transection of the jejunum the transected ends are apposed with 3–0 polydioxanone placed in a Lembert suture pattern and weighted with hemostatic mosquito forceps and a 50 g weight.

With owner consent, three jejunal segments were collected from eight horses, that were euthanized for reasons unrelated to intestinal pathology. Immediately after euthanasia, three mid‐jejunal segments 40 cm long with approximately 5–10 cm of mesentery were harvested. Segments were lavaged with room temperature Hartmanns solution (Vetivex 11, Dechra, Shrewsbury, UK), the lumen evacuated of ingesta, and the segments immersed in clean room temperature Hartmanns solution. All investigations were completed within 4 h of euthanasia. Two board‐certified surgeons (CMB and AMG) performed all intestinal harvesting, data collection, and anastomoses. Angle groups were evenly distributed between surgeons; the order of construction was randomly assigned. Prior to starting the resection and anastomosis, the mesenteric to antimesenteric width of the jejunum was measured by Vernier calipers (Figure 1). The intestine was not stretched during this process.

### 2.1 Anastomosis

**FIGURE 2:**
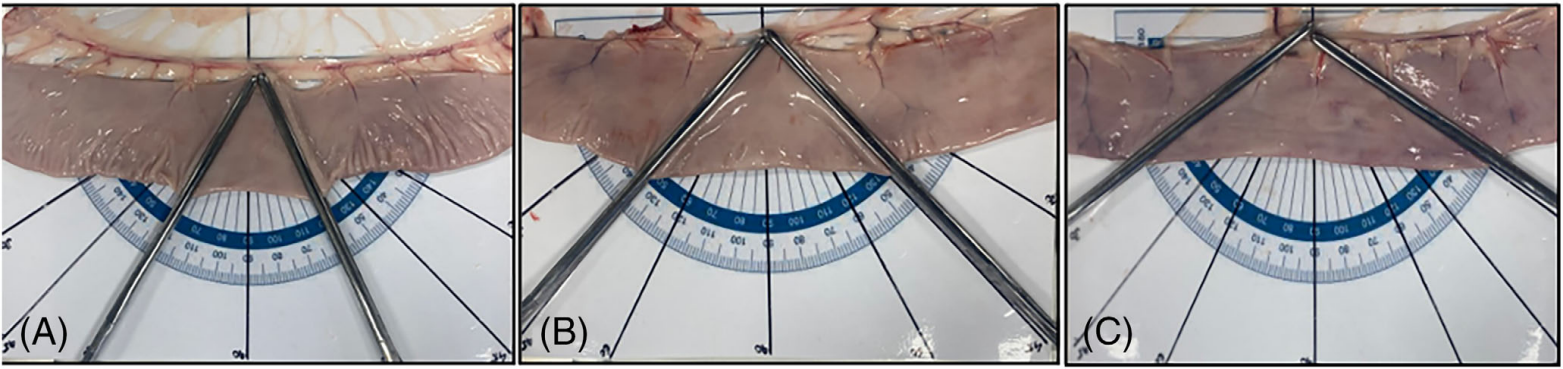
Images showing Doyen bowel clamps placed across cadaver sections of jejunum at various degrees of jejunal transection as demonstrated on the protractor image. (A) 60° of transection, (B) 45°of transection and (C) 30° of transection.

Jejunal segments were positioned flat on a steel tray customized with a template protractor with the angles 30, 45, and 60° emphasized (Figure 1). Each jejunal segment was randomly assigned to one group depending on the angle of transection, 30° (A30), 45° (A45) or 60° (A60). From each horse all three angles were tested but each 40 cm jejunal segment underwent only one anastomosis and each segment served as its own control. The mesenteric edge of the intestine was aligned to the horizontal line of the protractor and Doyen bowel clamps were clamped across the jejunum along either the 30, 45, or 60° lines, depending on group assignment (Figure 2). The remainder of the anastomosis construction largely follows a previously described method.^4^ In brief, a triangular wedge of jejunum was removed from the middle of each jejunal specimen using curved Metzenbaum scissors cutting the intestine along the Doyen bowel clamps. Prior to releasing the clamps the length of jejunal transection was measured with the Vernier calipers. The middle section of jejunum was discarded and the mesentery transected. All anastomoses were performed using 3–0 polydioxanone (Ethicon Inc., Somerville, New Jersey). Lembert stay sutures were inserted at the mesenteric and antimesenteric borders to appose the transected segments. To standardize jejunal tension the stay sutures were clamped to Halstead mosquito artery forceps with a 50 g weight suspended from the artery forceps equaling a combined weight of 73 g (Figure 1). A one‐layer interrupted modified‐Lembert pattern was used to construct the anastomosis.^4^ Suture bites were 8 mm wide and 8 mm apart as measured with Vernier calipers.^4^ The needle exited the tissue within 1 mm of the incised edge taking care to engage the submucosa with each bite. All suture lines started at the mesenteric edge, continued towards the antimesenteric border, and were repeated on the other side. All knots were secured with five throws. Additional sutures were placed if the distance between the sutures adjacent to the stay suture was greater than 8 mm. This ensured no gaps greater than 8 mm and a clinically acceptable anastomosis. The number of sutures placed was recorded. Anastomosis construction time, measured from placement of the first suture bite of the first stay suture until completion of the final knot in the anastomosis, was recorded.

### 2.2 Measurement of lumen size

**FIGURE 3:**
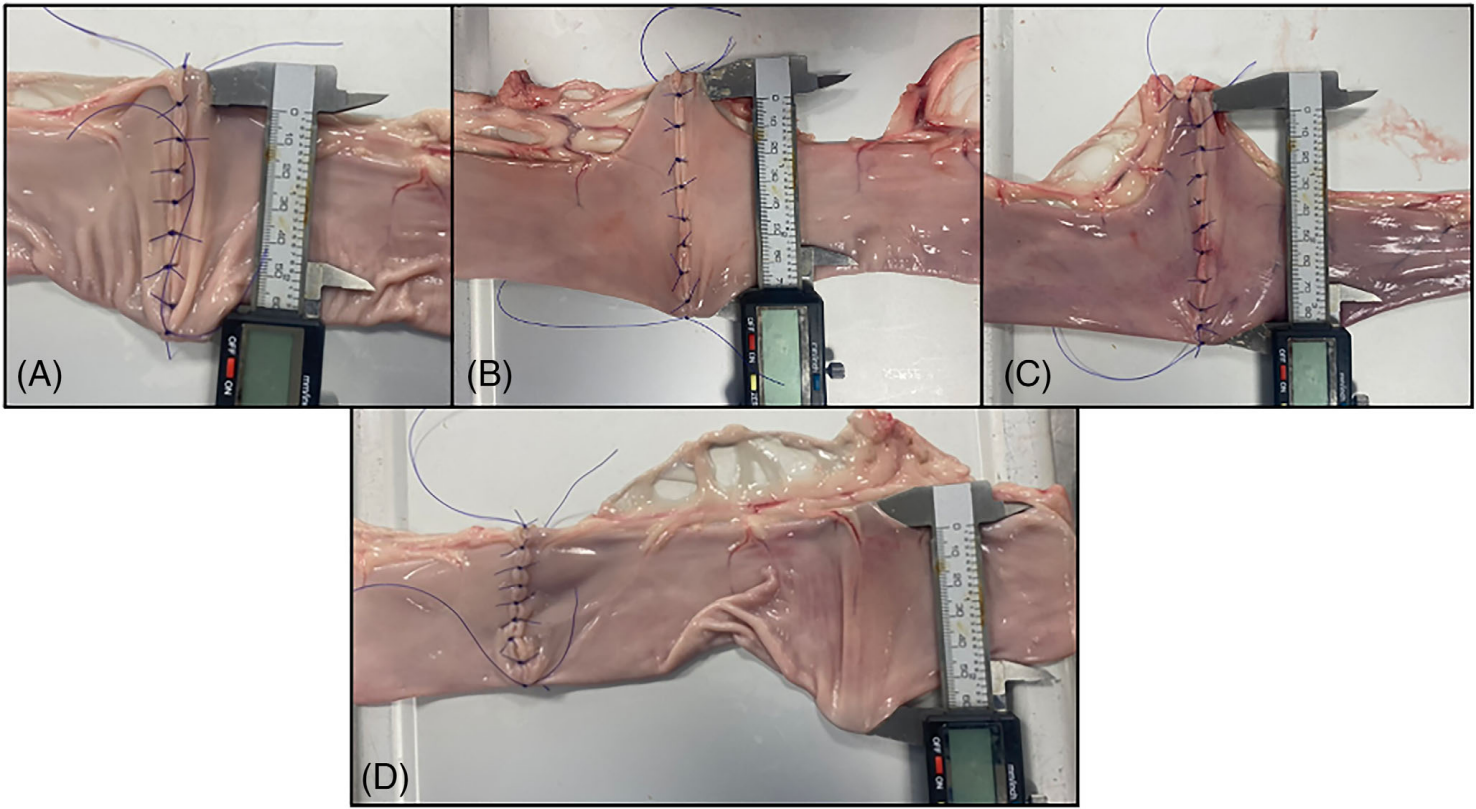
Vernier calipers placed within the lumen of the jejunum following transection and anastomosis construction at (A) 60° of transection, (B) 45° of transection, (C) 30° of transection and (D) 10 cm from the anastomosis site as a control section. The Vernier calipers are opened to maximal stretching and a measurement is recorded.

The protocol for measurement of lumen size follows a previously described method.^4^ In brief, as a control lumen size measurement, the lumen size of the same segment of jejunum was measured 10 cm from one side of the anastomosis. Subjective visual assessment was used to confirm that control sections were representative of the entire segment. Two full‐thickness 10 mm stab incisions were made on the mesenteric and antimesenteric margin of the jejunum and the forks of Vernier calipers were inserted into the jejunum lumen. The forks of Vernier calipers were opened until jejunal stretching was observed and clear resistance to further expansion was noted, the internal size was then recorded (Figure 3). To measure the lumen size of the anastomosis, two 10 mm full‐thickness stab incisions were made on the mesenteric and antimesenteric margin of the jejunum adjacent to the anastomosis. The forks of Vernier calipers were inserted across the anastomoses lumen and as before, the Vernier calipers were opened until jejunal stretching was observed and clear resistance to further expansion was noted, the internal size was then recorded (Figure 3). Each lumen size measurement at the control and anastomoses site was repeated three times over approximately 30 s. The anastomotic index was calculated as anastomotic stomal size/control stomal size × 100.^3^, ^4^ An anastomotic index >100 was considered an increase in lumen size over control and <100 was considered a reduction.

### 2.3 Statistical analysis

Descriptive analysis was performed in Excel (Microsoft Corporation, UK) and statistical analysis was performed in SPSS (IBM, Armonk, New York). Data were evaluated using Shapiro–wilk and Kolmogorov–Smirnov tests for normality. Due to the low number of data, Q‐Q plots were also generated to visually assess for normality. Results are reported as mean ± SD. Statistical analysis comparing mesenteric to antimesenteric widths of the unmanipulated intestine, jejunal transection length, time taken for anastomosis construction, number of sutures required, and AI between groups was performed using a one‐way ANOVA and post hoc Tukey HSD. Statistical significance was set at *p* < .05.

## 3 RESULTS

A total of 8 horses, six geldings and two mares ranging from 14 to 20 years old were used in the study. Breeds included were Pony (4), Warmblood (1), Warmblood cross Thoroughbred (1), Thoroughbred (1) and Connemara (1). Bodyweight ranged from 320 kg to 600 kg.

All data discussed were normally distributed. The resting mesenteric to antimesenteric width of the jejunal sections were not different between anastomoses groups A30, A45 or A60 (A30 mean: 41.56 ± 2.02; A45 mean: 45.75 ± 11.99; A60 mean: 43.88 ± 9.78; *p* = .762). Transected jejunal lengths were different between groups (*p* < .001). The mean transected jejunal lengths for A60, A45 and A30 were 54.8 cm (SD 13), 70.9 cm (SD 12.3) and 86 cm (SD 15.5), respectively. The difference in jejunal transection length was not different between A60 and A45 (*p* = .71) nor between A45 and A30 (*p* = .09) but it was different between A60 and A30 (*p* < .001).

The time taken to complete the anastomosis was shortest for the A60 and longest for the A30 group (Table 1), but was not different between anastomosis groups (*p* = .301). The mean number of sutures required to complete the anastomosis was lowest in group A60, and highest in group A30 (Table 1). The number of sutures required to complete the anastomosis was higher (*p* = .026) for A30 compared to A60 but was not different between A60 and A45 (*p* = .333) or between A45 and A30 (*p* = .371).

**TABLE 1:** Time for construction, number of sutures placed and AI of three different transection angles, (60, 45, and 30°) for end‐to‐end jejunojujenostomy anastomoses in equine cadaveric jejunal segments.

	Transection angle (degrees)			ANOVA comparison between groups (*p*‐value)			
	60	45	30	60 vs. 45 vs. 30	60 vs. 45	45 vs. 30	60 vs. 30
Time to complete	11 min 51 s ± 171 s	12 min 36 s ± 135 s	14 min 0 s ± 184 s	.301	.846	.574	.280
Number of sutures	16.88 ± 2.17	19.25 ± 3.24	21.50 ± 4.11	.034	.333	.371	.026
AI	93.37 ± 8.58	114.29 ± 9.61	135.07 ± 13.10	<.001	.002	.002	<.001

*Note*: The AI is the ratio between lumen size at the level of the anastomosis and that of an adjacent segment × 100. Results are reported as means and standard deviation.

Abbreviation: AI, anastomotic index.

**FIGURE 4:**
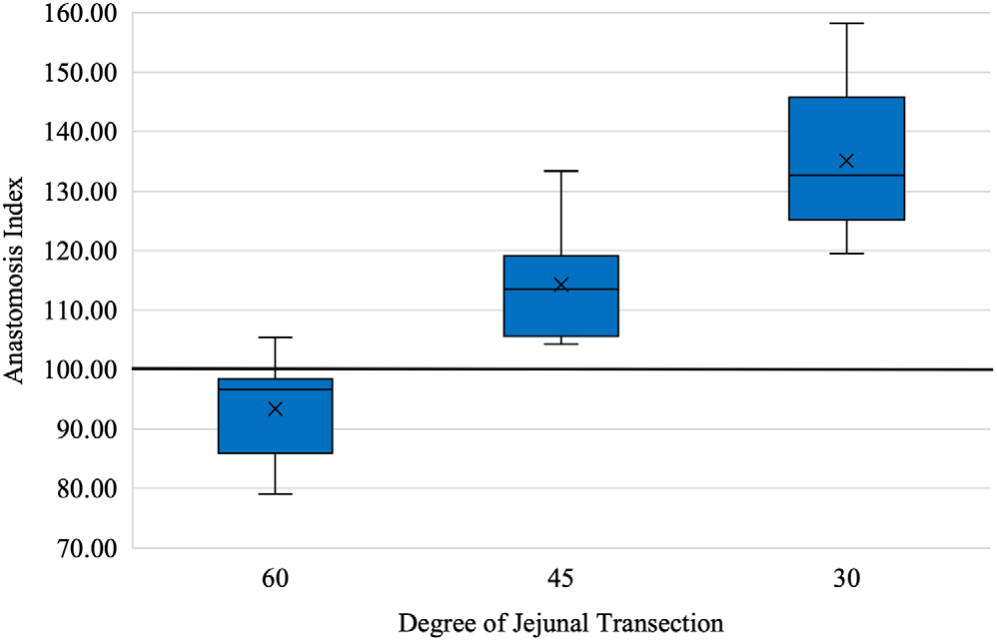
Box and whisker graph demonstrates the anastomosis index following 60, 45 or 30° jejunal transection and end‐to‐end jejunojejunostomy in an ex vivo cadaver study. The differences in the anastomosis index between groups were *p* < .002. The bold line indicates an anastomosis index of 100, data below this line indicates a reduction in lumen size compared to the control section; data above this line indicates an increase in lumen size compared to the control section.

The AI was different between all groups (*p* < .001). The mean AI for A60 was 93.37 (SD 8.58), the mean AI for A45 was 114.29 (SD 9.61) and the mean AI for A30 was 135.07 (SD 13.10). There was an increase in AI between A60 and A45 (*p* = .002) between A45 and A30 (*p* = .002) and between A60 and A30 (*p* < .001) (Table 1, Figure 4).

## 4 DISCUSSION

In line with our initial hypothesis, anastomosis lumen size increased as the transection angle decreased. Whilst the number of sutures required increased from A60 to A30, this difference was not significant for the A45 group. Construction time did not differ between groups. The results of this ex vivo cadaver study indicate that a 45° transection angle of the jejunum did not significantly increase construction time or the number of sutures but did significantly increase lumen size in all ex vivo specimens, compared to a 60° transection angle. The 30° transection angle provided the largest lumen size but required significantly more sutures.

The AI significantly increased as the transection angle decreased. This is to be expected as the length of transected jejunum increased with the decreasing transection angle. The currently recommended 60° transection angle produced a mean AI of less than 100, indicating a reduction in lumen size compared to the adjacent jejunum. This is in line with the majority of other studies assessing a single‐layer end‐to‐end jejunojejunostomy^3^, ^6^, ^15^, ^16^ and reflects the consequences of utilizing an inverting anastomosis pattern. Seitz‐Cherner et al.^4^ is the only publication to demonstrate an increase in anastomosis lumen size utilizing this anastomosis technique; however, this increase in AI was not identified in vivo and this study also found an increase in lumen size with other anastomosis suture patterns. The difference between our results and Seitz‐Cherner et al. is likely due to intersurgeon technique and variability in suture bite placement.^4^

The time to complete the anastomosis was not different between groups. This is important as increasing surgical time is associated with a reduction in survival^21^, ^22^, ^23^ and increased postoperative complications.^24^, ^25^, ^26^ However, the number of sutures required to complete the anastomosis was different between groups A30 and A60. Increasing suture material increases the risk of adhesion formation,^27^ therefore, reducing suture material may a desirable attribute in anastomosis construction. Although previous descriptions state that an inverting suture pattern will be less likely to create adhesions than an interrupted pattern where the knots are exposed,^27^ to the authors' knowledge, there are no reports of the number of sutures affecting adhesion formation in small intestinal surgery and this is an area for further research.

It is the author's opinion that there is sometimes confusion amongst colleagues and training residents as to where the transection angle should be measured. As described in Freeman et al.^1^ the angle of transection should be measured from the mesenteric border; however, should one measure transection angle from 90° (perpendicular) to the mesentery this would result in a wider 30° transection of the bowel. A 45° angle of transection eliminates uncertainty as to where to measure the transection angle.

One feature to note with an increasing transection angle is the creation of a triangular outpouching of the jejunum at the mesenteric border, as seen in Figure 3. This outpouching increased as the transection angle increased and likely contributed to the increase in AI in the A45 and A30 groups. Whilst this outpouching may appear to pose a risk of ingesta impaction, it is the author's clinical experience and opinion that when a 45° transection angle is utilized in clinical cases the muscle tone of the jejunum reduces this outpouching. Some surgeons have argued that this outpouching may be protective, as the anastomosis lumen size does change as a result of healing, muscle tone, inflammation, or fibrosis.^2^, ^4^, ^10^, ^18^, ^19^, ^20^ A balance between a lumen large enough to avoid constriction but not so large to result in an outpouching impaction needs to be considered.

As with all ex vivo studies there are associated limitations including factors related to anastomosis healing and the effect on the blood supply to the remaining jejunal ends. It should be acknowledged there is the possibility that a more severe reduction in lumen size may develop in vivo and postoperatively.^2^, ^4^, ^10^, ^18^, ^19^, ^20^ In addition, bursting strength was not evaluated in this study as bursting strength has been repeatedly evaluated with this suture material and pattern and the angle of transection was not considered to be a factor that would significantly affect bursting strength. Further limitations include the use of the calipers was not blinded and stretching could have been standardized with weights. Whilst additional sutures placed in the anastomosis were not recorded and may have affected stretching it was considered more important to construct clinically acceptable anastomoses. This study involved only a small sample size. A Cohen's D of 0.8 was utilized to establish a sample size; such a high Cohen's D accounts for only large differences in data. This data should therefore be considered preliminary and further investigation is required.

## 5 CONCLUSION

Results of this study indicate a 60° transection angle was insufficient to provide an anastomosis lumen equal to or greater than the adjacent jejunum. A 45° transection angle did not significantly increase surgical time or the number of sutures required but did result in an AI greater than 100 in all specimens. Although a 30° transection angle provided the largest AI it also significantly increased suture material. A larger ex vivo study and prospective case–control trial are required to assess the optimal transection angle. Further in vivo and clinical studies are required to evaluate this modification in technique.

## AUTHOR CONTRIBUTIONS

Baldwin CM, BVetMed (Hons), CertAVP (ESST) (EOS), AFHEA, Dip.ECVS, MRCVS and Gillen A, MA, MS, VetMB, CertAVP, DACVS (Large Animal), Dip.ECVS, FHEA, MRCVS: Contributed to the study design and execution, data analysis and interpretation, and preparation of the manuscript. All authors gave their final approval of the manuscript.

## CONFLICT OF INTEREST STATEMENT

The authors confirm there are no conflicts of interest.

## Data Availability

Anonymized data to support the findings from this study can be requested by contacting the corresponding author.
